# Ruptured Splenic Artery Aneurysms and the Use of an Adapted Fast Protocol in Reproductive Age Women with Hemodynamic Collapse: Case Series

**DOI:** 10.1155/2014/454923

**Published:** 2014-03-09

**Authors:** Hope T. Jackson, Silviu C. Diaconu, Patrick J. Maluso, Bruce Abell, Juliet Lee

**Affiliations:** Department of Surgery, George Washington University School of Medicine & Health Sciences, 2150 Pennsylvania Avenue, NW Suite 6B, Washington, DC 20037, USA

## Abstract

Nontraumatic symptomatic hypotension in all patients requires prompt diagnosis and appropriate treatment for optimum outcome. The female population specifically has an expanded differential diagnosis that should be considered when these patients present with hemodynamic collapse. While the most common causes of hypotension in pregnant patients are dehydration, ruptured ectopic pregnancy, and placental and uterine abnormalities, less common nonobstetrical etiologies such as hepatic rupture and ruptured abdominal and visceral artery aneurysms should also be considered. Splenic artery aneurysms are associated with high rates of mortality and in cases of pregnancy, maternal and fetal mortality. These high rates can be attributed to the asymptomatic nature of the aneurysm, rapid deterioration after rupture, and frequent misdiagnosis. In patients with hemodynamic collapse, the role of traditional imaging is limited mainly due to the critical condition of the patient. Bedside ultrasound has emerged as a diagnostic imaging resource in patients with undifferentiated hypotension and in patients with traumatic injuries. However, its use has not been studied specifically in the female population. We present two patients with ruptured splenic artery aneurysms, discuss the role of bedside ultrasound in their management, and introduce a new ultrasound protocol for use in reproductive age female patients with hemodynamic collapse.

## 1. Introduction

Nontraumatic symptomatic hypotension in any patient requires prompt diagnosis and appropriate treatment for optimum outcome. The female population has specific and unique causes of hypotension that should be evaluated. Severe dehydration and ruptured ectopic pregnancy are common etiologies of hypotension and, in known pregnant patients, placental abruption, placenta previa, uterine rupture, and pulmonary embolus should be considered [1]. Less common nonobstetrical etiologies such as hepatic rupture, ruptured abdominal aneurysm, and visceral artery aneurysms should also remain in the differential diagnosis. Misdiagnosis of these intra-abdominal sources of bleeding is common and brings potentially devastating outcomes.

In the diagnostic evaluation of unstable patients, the use of a CT scan, MRI, or angiography has a limited role primarily because of their time consuming nature. Ultrasound can be used to diagnose common obstetrical emergencies such as placental abruption, placenta previa, and uterine rupture [1]. Furthermore, several case reports comment on the use of bedside ultrasound to detect intra-abdominal free fluid to aid in the diagnosis of the less common causes of antepartum hemorrhage such as hepatic rupture and splenic artery aneurysm (SAA) rupture [2–5].

Focused Assessment with Sonography for Trauma (FAST) scan is well established in trauma literature and has been shown to detect as little as 100 mL of intra-abdominal fluid with 88% sensitivity [6]. However, little exists in terms of the use of ultrasound in hypotensive patients with no acute history of trauma. A few studies have explored the use of a bedside ultrasound protocol in the evaluation of emergency department (ED) patients with undifferentiated hypotension and have shown that the use of bedside ultrasound can help narrow the differential diagnosis and shorten the time to diagnosis [7–10]. However, these studies did not specifically focus on female patients.

In this case series, we present two patients, one with a known pregnancy and the other with unknown pregnancy status, who presented within the span of two weeks with ruptured splenic artery aneurysms. We then discuss the role of bedside ultrasound in their triage and surgical management and propose a new ultrasound protocol for use in female patients with hemodynamic collapse.

## 2. Case Examples

### 2.1. Case  1

**Figure 1:**
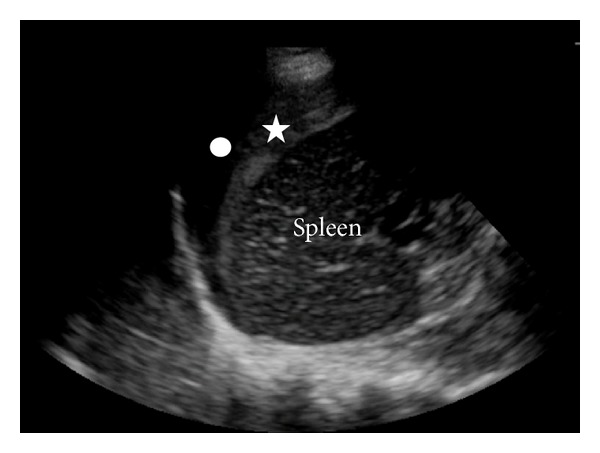
Left upper quadrant screenshot of bedside ultrasound performed in Case 1. The star indicates clotted blood adherent to the spleen and the circle indicates surrounding anechoic blood.

A 31-year-old woman, in her 35th week of pregnancy, presented to the ED after having witnessed seizure-like activity while sitting in a parked motor vehicle. Upon arrival by EMS, she was no longer having seizures but was noted to be unresponsive to questioning. Once in the ED, she was protecting her airway but was confused; her initial blood pressure and heart rate readings were 149/117 and 134, respectively. A fetal sonogram noted an intrauterine pregnancy and severe bradycardia with a heart rate in the 60s. With an initial presumed diagnosis of eclampsia, the obstetrical service was paged to the bedside emergently. Despite fluid resuscitation, the patient became hypotensive with systolic pressures in the 80s and continued altered mental status. A focused bedside abdominal ultrasound was performed and there was significant free fluid in bilateral upper quadrants (Figure 1). General surgery was immediately paged and the patient was taken emergently to the main surgical operating room for an exploratory laparotomy and cesarean section.

The patient's abdomen was explored revealing massive hemoperitoneum (approximately 4 liters) with a grossly normal liver in the right upper quadrant and brisk arterial bleeding in the left upper quadrant noted to be coming from a ruptured SAA at the lower pole of the spleen. A splenectomy was performed and the proximal splenic vein was noted to be markedly dilated with several collateral veins. The artery was noted to have a second aneurysm with three feeding vessels which were ligated and the second aneurysm was excluded.

Her postoperative course was uncomplicated and she was discharged home on postoperative day number 13. Though her infant was transferred to a tertiary children's hospital the day after delivery, he ultimately expired within the first days of life. The patient herself has done well and successfully carried another pregnancy to term without complication two years later.

### 2.2. Case  2

**Figure 2:**
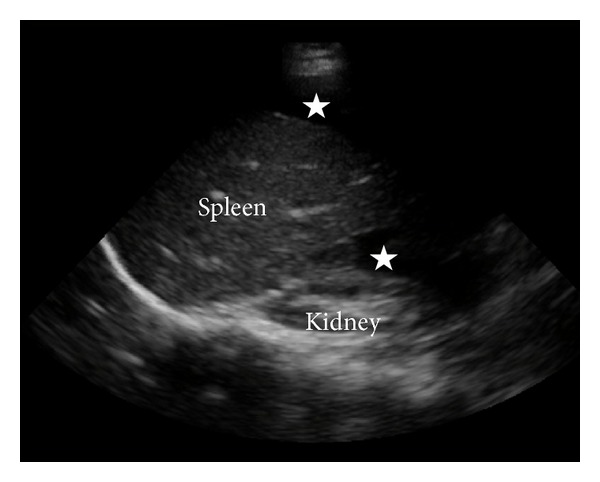
Left upper quadrant screenshot of bedside ultrasound performed in Case 2. The stars indicate surrounding anechoic blood.

A 28-year-old woman with no past medical or surgical history presented to the ED after the sudden onset of severe lower abdominal pain. Per EMS report, she was hypotensive at the scene (SBP 80s) but was conversant. Upon arrival in the ED, initial blood pressure and heart rate readings were 89/67 and 138 and she was noted to be diaphoretic and pale. While attempting to remove clothing, she collapsed onto the stretcher and became unresponsive. CPR and ACLS protocol were initiated as central venous access was established. Following approximately six minutes of CPR, a sinus tachycardia rhythm was achieved. At that time, focused bedside abdominal ultrasound revealed large amounts of free fluid in the abdomen and the patient was immediately taken to the operating room for an exploratory laparotomy with general surgery (Figure 2). Obstetrics was made aware of this patient in the event of an ectopic pregnancy and they were present in the operating room.

The patient's abdomen was explored revealing massive hemoperitoneum (greater than 5 liters) with a nongravid uterus, normal fallopian tubes, and ovaries without evidence of an ectopic pregnancy. The liver was grossly normal. In the left upper quadrant, brisk arterial bleeding was noted to be coming from a 1 cm opening in the splenic artery which was presumed to be a ruptured SAA. A splenectomy was performed and the remainder of the abdominal exploration was unremarkable.

Her postoperative course was only complicated by transient elevation in pancreatic enzymes that was managed nonoperatively with slow advancement of her diet. She was discharged home on postoperative day number 16 at her prehospitalization neurologic status.

## 3. Discussion

Splenic artery aneurysms (SAA) are the most common visceral artery aneurysms and third most common intra-abdominal aneurysms [11]. SAA are most commonly associated with pregnancy, and portal hypertension though essential hypertension, atherosclerosis and various congenital diseases have also been implicated [12].

Approximately 70% of ruptured SAA in pregnant patients are initially diagnosed as uterine rupture [13]. Although rare, the mortality for ruptures in nonpregnant patients is approximately 25–36% [14, 15]. This rate nearly doubles in pregnant patients (65–75%, >90% fetal mortality) and patients with portal hypertension (>50%) [16]. These high rates can be attributed to the asymptomatic nature of the aneurysm, rapid deterioration after rupture, and frequent misdiagnosis. Therefore, it is important for clinicians to quickly distinguish between obstetrical and nonobstetrical bleeding to appropriately triage patients for optimal surgical management.

The above case presentations underscore how focused bedside sonography can be effectively used in time-dependent situations to help elucidate a differential diagnosis and allow for optimal management plans in hypotensive female patients. In the case of our pregnant patient, without the use of the ultrasound, the patient could have easily been triaged to the obstetrical operating room for emergent cesarean with a presumed diagnosis of severe eclampsia. Similarly with our nonpregnant patient, the use of ultrasound allowed for the early involvement of a general surgeon, transfer to the main surgical operating room for laparotomy, and more efficient use of general surgery and obstetrical team resources. A 2009 review found that only 40% of the SAA reported cases (13 of 32) involved a general or vascular surgeon. The involvement of a general or vascular surgeon in these cases was found to be protective for the patient when a SAA rupture is diagnosed. Mortality increased in the group of patients with no general or vascular surgeon involved [17]. The early involvement of the appropriate surgical teams cannot be overemphasized and has particular importance in nontertiary, nontrauma centers, or community hospitals where surgeons may not be immediately available for consultation.

While ultrasound protocols have been suggested for the evaluation of a patient with undifferentiated hypotension, these protocols have not specifically focused on the female patients and their unique causes of symptomatic hypotension. We propose a protocol that extrapolates on the usual views of the FAST examination to differentiate between obstetrical and nonobstetrical causes of symptomatic hypotension and encourages the early involvement of the appropriate management teams.

**Figure 3:**
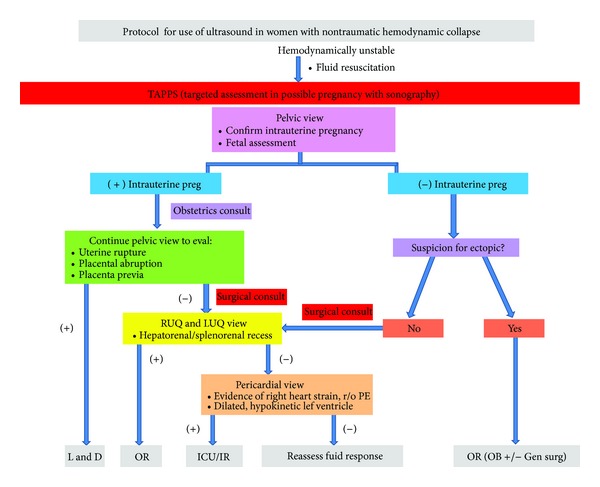
Targeted assessment in possible pregnancy with sonography (TAPPS). Ultrasound protocol for female reproductive age patients with non-traumatic hemodynamic collapse.

FAST is a bedside sonography examination performed for rapid assessment of a hemodynamically unstable trauma patient. The four views—subxiphoid (cardiac view), right upper quadrant (hepatorenal recess), left upper quadrant (splenorenal recess), and pelvic (bladder evaluation)—assess for pericardial and intraperitoneal fluid. In order to facilitate effective diagnosis and management, each view should be performed and interpreted in the context of collapse specific to the female population. We propose targeted assessment in possible pregnancy with sonography (TAPPS) as a new initial framework to help with the effective triage and management of these patients (Figure 3). We suggest performance of the pelvic view initially so that a patient with a known pregnancy can be quickly ruled in or out for an obstetrical cause of hemodynamic collapse. In a woman of child-bearing age but unknown pregnancy status, a pelvic view may quickly establish intrauterine pregnancy versus an ectopic pregnancy. While studies have shown transvaginal ultrasound to be superior to transabdominal ultrasound in the diagnosis of ectopic pregnancy (90% sensitivity, 99% specificity), these studies were not performed in patients with hemodynamic collapse [18]. The use of the transabdominal ultrasound in the ED has been shown to have 82% sensitivity and 92% specificity for evaluating the presence or absence of an intrauterine pregnancy [19]. Therefore, in the scenario of hemodynamic collapse, the use of transabdominal ultrasound to establish intrauterine pregnancy is likely the more time-effective and user-independent diagnostic study to perform. If an intrauterine pregnancy is not established and there is a suspicion of an ectopic pregnancy, the patient should be taken to the OR with the obstetrics service. Once an intrauterine pregnancy is established and the pelvic view also suggests a uterine rupture, placenta previa, or abruption, the patient should be immediately taken to labor and delivery. If the pelvic view is negative for these or in the case of a negative intrauterine pregnancy with no suspicion of an ectopic pregnancy, we recommend performing right and left upper quadrant views of the hepatorenal and splenorenal recesses, respectively. The likelihood of a hepatic or splenic etiology is high with either a positive right or left upper quadrant view and an immediate surgical consult should be initiated and the patient should be prepared for the operating room. If these views are negative, we would proceed with a pericardial view to evaluate for right heart strain suggestive of a pulmonary embolus (PE) or a hypokinetic dilated left ventricle suggestive of peripartum cardiomyopathy. The patient should be taken to the ICU if this view is positive or directly to interventional radiology in the setting of a massive PE. Further studies to evaluate the validity and effectiveness of the proposed protocol are needed and could lead to improved diagnosis and management of time-dependent conditions in this population.

## 4. Conclusion

The female patient of reproductive age has unique causes of hypotension that must be considered when evaluating patients who present with nontraumatic hypovolemic shock. Ectopic pregnancy, placental abnormalities, uterine rupture, and pulmonary embolus should be considered when evaluating these patients. However, as our case examples indicate, less common nonobstetrical etiologies such as ruptured visceral artery aneurysms should also be considered. Bedside ultrasound plays an important role in the diagnostic workup of these patients. While there are existing ultrasound protocols that target the workup of undifferentiated hypotension, this is the first protocol to our knowledge that combines the principles of known protocols to specifically target the unique causes of hypotension in this population of patients and encourage the early involvement of the appropriate management teams.
